# Supplementary material for: Short and Long Term Clinical and Immunologic Follow up after Bone Marrow Mesenchymal Stromal Cell Therapy in Progressive Multiple Sclerosis—A Phase I Study
Source: J Clin Med. 2019 Dec 2;8(12):2102. doi: 10.3390/jcm8122102 (PMC6947442; doi:10.3390/jcm8122102)
Supplement: Supplementary file 1 [file jcm-08-02102-s001.pdf]

Supplementary Tables
Iacobaeus, Kadri *et al.* Short and long term clinical and immunologic follow up after bone marrow mesenchymal stromal cell therapy in patients with progressive multiple sclerosis - a phase I study.

Table S1. Inclusion and exclusion criteria for the clinical MSC trial

Inclusion criteria

1. Diagnosis of Multiple Sclerosis (MS), with a subdiagnosis of
   a) Relapsing remitting MS with ≥ 1 relapse within the last 12 months, or ≥ 2 relapses or ≥ 1 MRI Gd+ lesion, within the last 24 months.
   b) Secondary progressive MS with increased EDSS of ≥ 1 point (or 0.5 point if baseline EDSS ≥ 5,5) within the last 12 months or if the patient has objective progress in the disease after one year with MS disease modifying treatments, (≥ 1 relapse or ≥ 1 new MRI Gd+ lesion).
   c) Primary progressive MS with increase in EDSS ≥1 point (or 0.5 point if EDSS ≥ 5,5 during the last year).
2. Age: 18 – 50 years.
3. MS disease duration of 2 – 20 years.
4. EDSS 3,0 – 7.

Exclusion criteria

1. RRMS not fulfilling inclusion criteria.
2. SPMS not fulfilling inclusion criteria.
3. PPMS not fulfilling inclusion criteria.
4. Active or chronic infection including infection with HIV1/2, Hepatitis B or C.
5. Treatment with any immunosuppressive therapy, including natalizumab and fingolimod, within 3 months prior to inclusion.
6. Treatment with interferon-beta or glatiramer acetate within the 30 days prior to inclusion.
7. Treatment with corticosteroids within the 30 days prior to inclusion.
8. Relapse occurred during the 60 days prior to inclusion.
9. Previous history of a malignancy other than basal cell carcinoma of the skin or carcinoma in situ that has been in remission for more than one year.

12\. Severe chronic illness with reduced life expectancy.
13\. Abnormal blood formation including myeodysplastic syndrome or other cytopenia.
12\. Pregnancy or risk or pregnancy (including patients that are unwilling to practice active contraception during the duration of the study).
13\. Renal failure or inability to undergo MRI examination.
14\. Inability to give written informed consent in accordance with research ethics board guidelines.

Table S2. Antibodies used for flow cytometry analyses

**Antigen**	**Clone**	**Company**
CD3	OKT3	Biolegend
CD4	OKT4	Biolegend
CD4	RPA-T4	BD Biosciences
CD4	OKT4	Biolegend
CD8	SK1	BD Biosciences
CD8	RPA-T8	Biolegend
CD14	HCD14	Biolegend
CD16	3G8	BD Biosciences
CD19	SJ25C1	BD Biosciences
CD19	SJ25C1	Biolegend
CD27	M-T271	Biolegend

CD31	WM59	Biolegend
CD34	561	Biolegend
CD45RA	HI100	Biolegend
CD45	2Di	Biolegend
CD55	JS11	Biolegend
CD56	5.1H11	Biolegend
CD56	NCAM	Biolegend
CD59	P282 (H19)	Biolegend
CD73	TY/11.8	Biolegend
CD90	5E10	Biolegend
CD105	43AJ	Biolegend
Anti-HLA ABC	W6/J2	Biolegend
HLA DR	L243	Biolegend
Foxp3	FJK-16s	Ebiosciences
LIVE/DEAD™ Fixable Dead Cell Stain		Invitrogen

Table S3. KEGG analysis

**+2 h data miRNA DIANA**			
**KEGG pathway**	**p-value**	**linked genes**	**miRNAs involved**
Proteoglycans in cancer	1.93146484573e-15	157	17
Cell cycle	1.12178094013e-07	98	15
Glioma	1.12178094013e-07	54	16
Hippo signaling pathway	1.05479502727e-06	110	18
Prion diseases	4.90460086806e-06	26	15
Hepatitis B	4.90460086806e-06	107	17
Pathways in cancer	4.90460086806e-06	275	18
mTOR signaling pathway	1.48337263916e-05	52	16
Renal cell carcinoma	1.48337263916e-05	55	16
Colorectal cancer	1.48337263916e-05	52	17
TGF-beta signaling pathway	1.84135622353e-05	62	15
Adherens junction	2.21609601208e-05	58	15
Prostate cancer	2.21609601208e-05	72	17
Non-small cell lung cancer	3.27997769701e-05	46	15
Pancreatic cancer	4.20885722089e-05	55	15
Chronic myeloid leukemia	8.02829175019e-05	60	15
Glycosaminoglycan biosynthesis - keratan sulfate	8.23787583371e-05	13	13
p53 signaling pathway	0.000258063209469	56	16
Viral carcinogenesis	0.000273944587532	140	16
Neurotrophin signaling pathway	0.000273944587532	91	16
Fatty acid elongation	0.000504403225555	17	10
Insulin signaling pathway	0.000706164162648	104	17
Thyroid cancer	0.000768194622121	25	13
Signaling pathways regulating stem cell pluripotency	0.000768194622121	100	15
Oocyte meiosis	0.000768194622121	83	16
MAPK signaling pathway	0.000768194622121	175	18
Acute myeloid leukemia	0.00078059807844	46	15
HTLV-I infection	0.00078059807844	179	17
Endometrial cancer	0.000833022125462	42	17

Bacterial invasion of epithelial cells	0.00160352770125	58	15
Central carbon metabolism in cancer	0.00203755145217	52	15
FoxO signaling pathway	0.00224525514518	96	16
Protein processing in endoplasmic reticulum	0.00224525514518	120	18
Endocytosis	0.00259393895211	139	17
Melanoma	0.0030113784351	53	15
ErbB signaling pathway	0.0030113784351	67	16
Wnt signaling pathway	0.00357473545519	98	16
TNF signaling pathway	0.00381123196942	78	16
Thyroid hormone signaling pathway	0.00489631689307	88	17
Regulation of actin cytoskeleton	0.00635310056358	143	16
Estrogen signaling pathway	0.00635310056358	73	17
Axon guidance	0.00707652630451	87	17
Shigellosis	0.00776654116275	47	15
RNA transport	0.00776654116275	117	18
HIF-1 signaling pathway	0.00776654116275	76	18
Progesterone-mediated oocyte maturation	0.00776654116275	65	14
Steroid biosynthesis	0.009438210104	15	10
Bladder cancer	0.0143747328282	31	14
Transcriptional misregulation in cancer	0.0146794734164	124	16
Long-term potentiation	0.0159868077105	50	17
Glycosphingolipid biosynthesis - lacto and neolacto series	0.0204462018285	18	10
Focal adhesion	0.0204462018285	139	18
Vibrio cholerae infection	0.0206424519226	41	16
One carbon pool by folate	0.0220927206966	15	14
Lysine degradation	0.027889294735	34	11
Ubiquitin mediated proteolysis	0.0294850122424	94	17
N-Glycan biosynthesis	0.0300426140281	35	15
Glycosaminoglycan biosynthesis - chondroitin sulfate / dermatan sulfate	0.032436352375	13	9
SNARE interactions in vesicular transport	0.0328772489673	26	10
DNA replication	0.0328772489673	26	12
Lysosome	0.0348345638075	82	16
Epithelial cell signaling in Helicobacter pylori infection	0.0348651125163	49	14
Legionellosis	0.0390893475659	41	15
Salmonella infection	0.0396716729584	60	16
T cell receptor signaling pathway	0.0415203407918	73	17
Sphingolipid signaling pathway	0.0447796927143	81	16
Prolactin signaling pathway	0.0447796927143	50	16
Small cell lung cancer	0.0463983364936	60	17
**List of 20 miRNAs analysed**	hsa-miR-193a-5p		
	hsa-miR-34a-5p		
	hsa-miR-365a-3p		
	hsa-miR-584-5p		
	hsa-miR-100-5p		
	hsa-miR-151a-3p		
	hsa-miR-192-5p		
	hsa-miR-215-5p		
	hsa-miR-335-5p		
	hsa-miR-155-5p		

	hsa-miR-543		
	hsa-miR-15b-5p		
	hsa-let-7d-3p		
	hsa-miR-126-3p		
	hsa-miR-140-3p		
	hsa-miR-374b-5p		
	hsa-miR-16-5p		
	hsa-miR-375		
	hsa-miR-30d-5p		
	hsa-miR-362-3p		
**+3 days data miRNA DIANA**			
ECM-receptor interaction	7.04032753998e-36	25	3
Fatty acid biosynthesis	1.05652307603e-13	4	3
Viral carcinogenesis	1.28462356474e-12	66	3
Hippo signaling pathway	9.21300711178e-09	40	3
Proteoglycans in cancer	3.33067650665e-08	52	3
Lysine degradation	4.31260460545e-08	16	3
p53 signaling pathway	2.3296081692e-05	26	3
Protein processing in endoplasmic reticulum	4.12518655228e-05	52	3
PI3K-Akt signaling pathway	0.000567675232578	85	3
Adherens junction	0.000567675232578	23	3
Huntington's disease	0.00115979224426	41	3
Colorectal cancer	0.00115979224426	20	3
Pantothenate and CoA biosynthesis	0.0021267733925	6	3
Hepatitis B	0.0021267733925	39	3
Central carbon metabolism in cancer	0.0021267733925	20	3
Bacterial invasion of epithelial cells	0.00296312178688	22	3
Glycosaminoglycan biosynthesis - chondroitin sulfate / dermatan sulfate	0.00310855745509	4	3
Focal adhesion	0.00360598326156	54	3
FoxO signaling pathway	0.00526871470309	37	3
Shigellosis	0.00526871470309	18	3
Thyroid hormone signaling pathway	0.00673089779958	30	3
Glioma	0.00673089779958	19	3
Cell cycle	0.006892240036	36	3
Pathways in cancer	0.006892240036	88	3
Endocytosis	0.0113016775271	49	3
Fatty acid metabolism	0.0115997644264	8	3
Small cell lung cancer	0.0115997644264	26	3
Prostate cancer	0.0122463955908	27	3
mTOR signaling pathway	0.0138050001093	20	3
Estrogen signaling pathway	0.0149409287564	25	3
Steroid biosynthesis	0.0158059744152	4	2
Pathogenic Escherichia coli infection	0.0158059744152	18	3
Renal cell carcinoma	0.0207561045355	18	3
Endometrial cancer	0.0207561045355	15	3
Amoebiasis	0.0224785695571	24	3
Bladder cancer	0.0236949972267	14	3
**List of 4 miRNAs analysed**	hsa-miR-133a-3p		
	hsa-miR-375		
	hsa-miR-143-3p		
	hsa-miR-29a-3p		

Table S4. Adverse events during MSC infusion and follow up of 48 weeks

	Number of patients affected
Facial rash during infusion	1
Fracture tibia, accidental fall	1
Urinary infection	2
Sinusitis	1
Pneumonia	1
Pseudorelapse (from previous ON)	1
Depressive symptoms	2
Pulmonary embolism	1
Relapse	1
